# Transfusion-transmitted severe *Plasmodium knowlesi* malaria in a splenectomized patient with beta-thalassaemia major in Sabah, Malaysia: a case report

**DOI:** 10.1186/s12936-016-1398-z

**Published:** 2016-07-12

**Authors:** Elspeth M. Bird, Uma Parameswaran, Timothy William, Tien Meng Khoo, Matthew J. Grigg, Ammar Aziz, Jutta Marfurt, Tsin W. Yeo, Sarah Auburn, Nicholas M. Anstey, Bridget E. Barber

**Affiliations:** Infectious Diseases Society Sabah Menzies School of Health Research Clinical Research Unit, Kota Kinabalu, 88586 Sabah Malaysia; Queen Elizabeth Hospital Clinical Research Centre, Kota Kinabalu, 88586 Malaysia; Jesselton Medical Centre, Kota Kinabalu, 88300 Sabah Malaysia; Intensive Care Unit, Queen Elizabeth Hospital, Kota Kinabalu, 88586 Sabah Malaysia; Menzies School of Health Research and Charles Darwin University, PO Box 41096, Casuarina, 0810 NT Australia; Lee Kong Chian School of Medicine, Nanyang Technological University, Singapore, 639798 Singapore

**Keywords:** *Plasmodium knowlesi*, Severe malaria, Transfusion-transmitted malaria, Malaria transmission, Beta thalassemia, Splenectomy, PCR, Sabah, Borneo

## Abstract

**Background:**

Transfusion-transmitted malaria (TTM) is a well-recognized risk of receiving blood transfusions, and has occurred with *Plasmodium falciparum, Plasmodium vivax, Plasmodium ovale*, and *Plasmodium malariae.* The simian parasite *Plasmodium knowlesi* is also known to be transmissible through inoculation of infected blood, and this species is now the most common cause of malaria in Malaysia with a high rate of severity and fatal cases reported. No confirmed case of accidental transfusion-transmitted *P. knowlesi* has yet been reported.

**Case presentation:**

A 23-year old splenectomized patient with beta thalassaemia major presented with fever 11 days after receiving a blood transfusion from a pre-symptomatic donor who presented with knowlesi malaria 12 days following blood donation. The infection resulted in severe disease in the recipient, with a parasite count of 84,000/µL and associated metabolic acidosis and multi-organ failure. She was treated with intravenous artesunate and made a good recovery. Sequencing of a highly diverse 649-base pair fragment of the *P. knowlesi* bifunctional dihydrofolate reductase-thymidylate synthase gene (*pkdhfr*) revealed that the recipient and donor shared the same haplotype.

**Conclusions:**

This case demonstrates that acquisition of *P. knowlesi* from blood transfusion can occur, and that clinical consequences can be severe. Furthermore, this case raises the possibility that thalassaemic patients, particularly those who are splenectomized, may represent a high-risk group for TTM and severe malaria. With rising *P. knowlesi* incidence, further studies in Sabah are required to determine the risk of TTM in order to guide screening strategies for blood transfusion services.

## Background

Transfusion-transmitted malaria (TTM) was first described in 1911 [1] and remains an important public health problem. In non-endemic countries, stringent screening processes have led to a very low incidence, with less than one case occurring per one million donations [2], however, clinical consequences can be severe due to lack of background immunity in the population [2, 3]. In endemic countries, the incidence of TTM is substantially higher due to a high prevalence of parasitaemia among blood donors; a systematic review in sub-Saharan Africa reported a median prevalence of parasitaemia among blood donors of 10.2 % (range 2–55 %) [4]. In endemic countries outside sub-Saharan Africa, malaria prevalence among blood donors is generally lower [5, 6], however risk of acquiring malaria from infected blood may be higher due to the lower immunity of recipients.

TTM has been reported to occur as a result of infection with *Plasmodium falciparum*, *Plasmodium vivax*, *Plasmodium malariae*, and *Plasmodium ovale* [7, 8], and one probable case of transfusion-transmitted *Plasmodium knowlesi* has been reported [9]. In Sabah, Malaysian Borneo, the overall incidence of clinical malaria is low, and population immunity likely minimal, however human cases of *P. knowlesi* are increasing, with the species now the most common cause of malaria in Sabah [10]. *Plasmodium knowlesi* has a 24-hour erythrocytic life cycle, and high parasitaemias can develop rapidly. In adults, the risk of severe disease is at least as high as that of *P. falciparum* [11], and fatal cases have been reported [10, 12–18]. Transfusion-transmitted *P. knowlesi* may therefore constitute a significant risk in knowlesi-endemic areas such as Sabah.

Current screening practices in Sabah include a pre-donation questionnaire to exclude donors with history of fever in the previous 7 days, and microscopic examination of a thick and thin blood film for malaria parasites. Microscopy, however, fails to detect parasitaemias <50/µL [19], and for *P. falciparum* it has been established that as few as ten parasites per unit of red blood cells are sufficient to transmit infection [20]. Screening with microscopy does not therefore eliminate the risk of TTM. Recently, the first reported Malaysian case of TTM was described, involving a child in West Malaysia infected with *P. vivax* from a donor recently returned from Myanmar [21]. Although *P. knowlesi* is well known from early experimental and neurosyphilis studies to be transmissible through inoculation of infected blood [22, 23], no confirmed case of accidental transfusion-transmitted *P. knowlesi* has yet been reported.

This report describes a case of severe transfusion-transmitted *P. knowlesi* infection in a splenectomized thalassaemic patient, acquired from a pre-symptomatic donor who presented with knowlesi malaria soon after donation.

## Case presentation

A 23-years old female presented to Pitas District Hospital in northeast Sabah, Malaysia, with a 5-day history of fever, rigours, headache, and dizziness. She lived with her family in a malaria-endemic village surrounded by palm oil plantations, where long-tailed macaques were regularly seen. She had a history of beta thalassaemia major, diagnosed at 9 months of age and requiring splenectomy at age 13 years. She received iron chelation and second monthly blood transfusions at her local hospital. Her last blood transfusion had been 16 days prior to presentation, and her post-transfusion haemoglobin was 9.8 g/dL. She had recently commenced oral hypoglycaemic agents for type 2 diabetes mellitus.

On presentation at the district hospital her vital signs were within normal range, including an oxygen saturation of 98 %. Her haemoglobin was 8.7 g/dL, white cell count 48 × 10^3^/µL and platelet count 129 × 10^3^/µL. She was admitted with a provisional diagnosis of symptomatic anaemia. Later that day she developed respiratory distress with an oxygen saturation of 50 % on room air, improving to 72 % on high-flow oxygen, and she was transferred to a tertiary referral hospital in the state capital, Kota Kinabalu. On arrival her temperature was 38.6 °C, heart rate 139 beats/min, blood pressure 116/69 mm Hg, and oxygen saturation 93 % on high-flow mask with a respiratory rate of 36 breaths per minute. A blood film taken on arrival was positive for malaria parasites resembling *P. knowlesi*, with a parasite count of 84,000 parasites/µL. Further investigation revealed worsening anaemia (haemoglobin 6.0 g/dL) and further elevation of the white blood cell count (61 × 10^3^/µL), in addition to metabolic acidosis (lactate 16 mmol/L, bicarbonate 8.4 mmol/L, pH 7.14), hyperbilirubinemia (80 µmol/L), acute kidney injury (creatinine 184 µmol/L), increased liver transaminases (alanine transaminase 181 U/L and aspartate transaminase 308 U/L), and hypoglycaemia (blood glucose 2.5 mmol/L) (Table 1). Chest radiograph was initially unremarkable, however, on day 1 showed mild bilateral interstitial infiltrates. She was diagnosed with severe knowlesi malaria, admitted to the intensive care unit, and commenced on intravenous artesunate, intravenous antibiotics (initially piperacillin/tazobactam, then ceftriaxone) and inotropic support; she was transfused two units of whole blood. She made a good recovery, and on day 2 after completing three doses of intravenous artesunate, she was changed to oral artemether-lumefantrine (Riamet^**®**^) for 3 days and discharged from the intensive care unit. Her blood film was negative for malaria parasites on day 3, and she was discharged from hospital on day 9. Dengue NS1 antigen and blood cultures taken prior to antibiotics were negative. PCR of her initial blood sample using previously described methods [24] confirmed *P. knowlesi*.

**Table 1:** Laboratory investigations

	Day 0^a^	Day 1	Day 2	Day 3	Day 4	Day 5	Day 7
Parasite count (parasites/µL)	84,000	22,667	313	0	0	0	0
Haemoglobin (g/dL)	6.0	11.7^b^	9.9	9.7	9.0	7.4	11.9^b^
Haematocrit (%)	17.9	33.4^b^	27.6	27.2	24.9	20.6	34.0^b^
White blood cells (×10^3^/µL)	61	27	28	26	24	20	26
Platelets (×10^3^/µL)	96	70	36	83	126	191	413
Creatinine (µmol/L)	184	127	52	63	63	48	60
Urea (mmol/L)	7.7	11.3	7.6	7.5	4.7	2.8	202
Sodium (mmol/L)	125	129	127	126	128	132	129
Potassium (mmol/L)	2.6	4.5	4.1	4.0	3.8	2.9	4.0
Bilirubin (µmol/L)	80	110	93	63	48	41	37
Lactate (µmol/L)	16	4.8	1.3				
Bicarbonate (mmol/L)	8.4	19.7	16.5				
Alanine transferase (IU/L)	181	236	168	136	83	70	55
Aspartate transferase (IU/L)	308	322	149	108	78	91	75
Glucose (mmol/L)	2.5			10.8			
Prothrombin time (seconds)	37.4	18.5	15.7				
APTT (seconds)	78.7	51.8	42.7				

*APTT* activated partial thromboplastin time

^a^Investigation results from referral hospital

^b^Post blood transfusion

## Donor

In view of the patient’s blood transfusion 16 days prior to admission, the possibility of transfusion-acquired *P. knowlesi* was investigated. The blood bank was contacted for information regarding the donor, the donor was informed, and donation records were obtained from the blood bank. Medical records of the donor were subsequently retrieved from Pitas District Hospital where he had been admitted, and reviewed for clinical and laboratory details. An EDTA blood sample was retrieved from the State Public Health Laboratory, where it had been sent for *Plasmodium* PCR.

The blood donor was a 51-years old male farmer who lived in a malaria-endemic village 3 h drive from the patient’s village, and who had donated blood 4 days prior to the patient’s blood transfusion. He was well on the day of the blood donation, and a screening blood film was negative for malaria parasites. Twelve days following the donation however, he was admitted to the same district hospital with a 5-day history of fever, chills, headache, nausea, and malaise. His blood film was reported as *P. falciparum* ‘2+’ (indicating 40-400 parasites/µL). He was treated with oral artesunate and mefloquine (Artequin^**®**^) for uncomplicated malaria, and was discharged after 4 days. PCR performed on an admission EDTA blood sample confirmed *P. knowlesi*.

## Molecular analysis

**Fig. 1:**
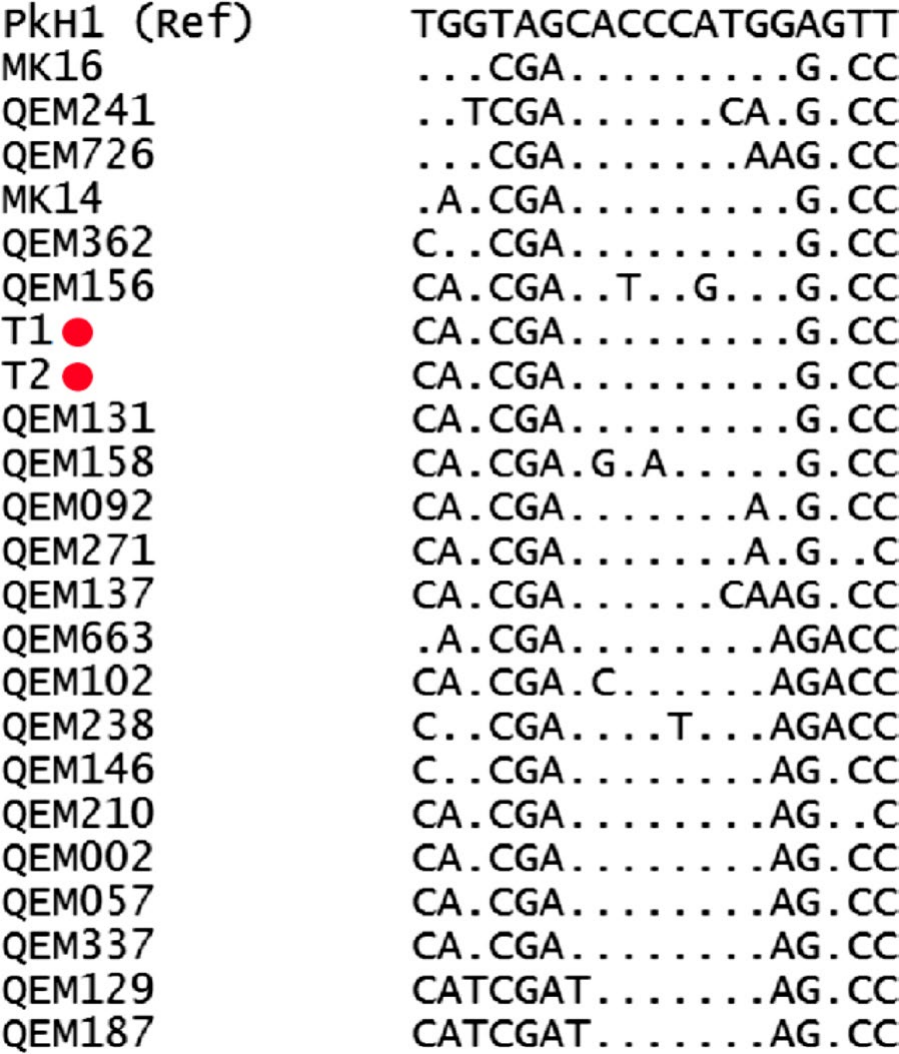
Sequence polymorphisms in the *pkdhfr* gene fragment in *Plasmodium knowlesi* infections in patients from the transfusion recipient’s district, including the transfusion recipient and donor (T1 and T2). Nineteen polymorphic sites are presented: from *left to right* representing nucleotide positions 36, 90, 101, 135, 192, 240, 252, 271, 288, 386, 418, 429, 446, 459, 495, 528, 570, 600 and 636 with numbering beginning from the first nucleotide of the start codon of *pkdhfr* in the *P. knowlesi* H strain (PkH1) reference sequence (GeneDB version 2015-06-18). *Dots* represent identical nucleotide residues

Molecular finger-printing of the patient and donor infections was undertaken using sequence data generated on a 649-base pair fragment of the *P. knowlesi* bifunctional dihydrofolate reductase-thymidylate synthase gene (*pkdhfr*, PKNH_0509600), which has been demonstrated to be highly diverse in Sabah [25]. DNA extraction and *pkdhfr* sequencing was conducted using the methods described by Grigg et al. [25]. Additional *pkdhfr* sequence data on 21 *P. knowlesi* infections sourced from the same district were retrieved from Grigg et al. to assess the local *pkdhfr* diversity. Analysis using DnaSP (version 5.10.01) confirmed high diversity amongst the 22 baseline samples. A total of 18 distinct haplotypes were observed, with haplotype diversity estimated at 0.978 (i.e., approximately 2 % probability that two independent infections randomly selected from the population would have identical haplotypes by chance alone). The patient and donor infections shared the same haplotype along with one other infection (QEM131) (Fig. 1).

## Discussion

This report describes a case of severe transfusion-transmitted knowlesi malaria, the occurrence of which may have implications for blood transfusion practices in knowlesi-endemic areas. *Plasmodium knowlesi* is the most common cause of malaria in Malaysia [10, 26, 27], and the species may cause severe and fatal disease [10, 12]. Cases of transfusion-transmitted falciparum malaria can rapidly be fatal in susceptible individuals [28]. Transfusion-transmitted *P. knowlesi* may represent a significant risk in endemic areas, particularly in those at high risk, such as the asplenic thalassaemic patient described in this report.

The risk of being exposed to *P. knowlesi* through blood transfusion in Sabah is difficult to estimate. Although no cross-sectional prevalence survey has yet been reported in Sabah, a recent molecular-based survey of individuals residing in households or villages of symptomatic malaria patients in northeast Sabah found that in this population the prevalence of asymptomatic *P. knowlesi* infection was 6.9 % [29]. In a recent study in Ghana, where 55 % of blood donors were parasitaemic when tested by PCR, the incidence of TTM in non-parasitaemic recipients receiving parasitaemic blood was around 14–28 % [30]. The risk of acquiring malaria from infected blood may be higher in Sabah where immunity of the population is likely to be low.

Beta thalassaemia has been shown to be a risk factor for other severe infections, with increasing risk associated with duration of thalassaemia, number of transfusions, previous splenectomy, and receiving the iron chelator deferoxamine [31]. Additional factors thought to contribute to infection risk include anaemia, reticulo-endothelial dysfunction resulting from iron overload and haemolysed erythroblasts, and altered immune responses [32]. The incidence of TTM among thalassaemic patients in endemic countries is difficult to quantify, as recipients frequently have pre-existing parasitaemia [20]. In a study in Sri Lanka, a higher frequency of *P. vivax* antibodies was found in thalassaemia patients compared to age-matched controls, with frequency particularly high in splenectomized patients [33]. An Indian study demonstrated post-transfusion malaria occurring in 6.4 % of beta thalassaemia patients who received repeated blood transfusion [34]. Malaysia has a high prevalence of thalassaemia; in 2009 there were 4541 registered thalassaemia patients, of whom 3310 were transfusion-dependent with either beta thalassaemia major or HbE beta thalassaemia. The state of Sabah has the highest number of registered thalassaemia cases, accounting for 28 % of the total cases in Malaysia [35]. TTM among the beta thalassaemia population in malaria-endemic regions of Sabah may be under-reported and requires further investigation.

In addition to being a possible risk factor for TTM, the lack of a spleen has been associated with severe malaria following infection with *P. falciparum* [36], and severe knowlesi malaria in splenectomized patients has also been reported [11, 37]. High parasite counts and severe disease, as occurred in this case, usually occur in older patients, being relatively uncommon among young patients with knowlesi malaria [11]. Over 100 cases of severe knowlesi malaria have now been reported [10–17, 37–44]; to our knowledge, only 6 cases have been reported in patients <30 years old [11, 13, 37], with only 2 of these having more than one severity criteria [13, 37]. Notably, the only previous report of multi-organ failure in a patient <30 years old occurred in a splenectomized patient [37]. It is therefore possible that in this 23-years old patient the lack of spleen contributed to her severe disease. Furthermore, direct inoculation of *P. knowlesi*-infected blood may have contributed to the development of severe disease; early studies from the neurosyphilis era demonstrated increased virulence of infection with serial blood passage through humans [45, 46], and more recently in a murine model parasite, virulence was found to be modified by vector transmission [47].

To reduce the risk of TTM in endemic countries the World Health Organization recommends several strategies, including donor selection and deferral, and/or screening of all donated blood for malaria parasites [48]. However, current screening strategies are associated with significant limitations. In Sabah, microscopy is used as the current screening policy, however it has poor sensitivity for low parasitaemias [29], and hence will fail to detect a significant proportion of sub-clinical infections, as occurred in the current case. Rapid diagnostic tests are also insufficiently sensitive, particularly for the diagnosis of knowlesi malaria [49, 50], and PCR, although sensitive, is limited by expense. Loop-mediated isothermal amplification (LAMP) assays have been developed recently and offer potential as a low–cost sensitive screening tool, but have been evaluated in only small numbers of patients [51–53]. Larger studies are required to assess the potential of this method for screening donated blood for malaria parasites.

An additional strategy proposed to prevent TTM in endemic countries is default malaria treatment of transfusion recipients [48], although the feasibility of this option is limited by the cost of artemisinin-based combination therapy. Moreover, in a country of low prevalence such as Sabah, this strategy would result in significant over-treatment of recipients. Recently, a whole blood pathogen reduction treatment was shown to reduce the incidence of TTM in addition to other transfusion-transmitted pathogens, demonstrating the potential of this technology to enhance safety of blood transfusions in malaria-endemic regions [54].

## Conclusion

This case demonstrates that acquisition of *P. knowlesi* through blood transfusion may occur, and that clinical consequences can be severe. It is possible that thalassaemic and/or splenectomized individuals constitute a particular risk group for TTM, and may be at high risk of severe disease. With a rising incidence of knowlesi malaria, further studies are required to determine the prevalence of malaria among blood donors in Sabah, and the risk of TTM among recipients, in order to guide screening strategies for blood transfusion services in the region. Finally, clinicians should be aware of the risk of TTM in any patient having received a blood transfusion, with post-transfusion febrile illnesses appropriately investigated.

## Abbreviations

*pkdhfr*: *P. knowlesi* bifunctional dihydrofolate reductase-thymidylate synthase gene
LAMP: loop-mediated isothermal amplification
TTM: transfusion transmitted malaria
PCR: polymerase chain reaction
